# TReatIng Urinary symptoms in Men in Primary Healthcare using non-pharmacological and non-surgical interventions (TRIUMPH) compared with usual care: study protocol for a cluster randomised controlled trial

**DOI:** 10.1186/s13063-019-3648-1

**Published:** 2019-09-02

**Authors:** Jessica Frost, J. Athene Lane, Nikki Cotterill, Mandy Fader, Lucy Hackshaw-McGeagh, Hashim Hashim, Margaret Macaulay, Stephanie J. MacNeill, Sian Noble, Jonathan Rees, Matthew J. Ridd, Luke Robles, Gordon Taylor, Jodi Taylor, Marcus J Drake, Jo Worthington

**Affiliations:** 1 Bristol Randomised Trials Collaboration (BRTC), Population Health Sciences, Bristol Medical School, University of Bristol, Canynge Hall, 39 Whatley Road, Bristol, BS8 2PS UK; 2 Bristol Urological Institute, Level 3, Learning and Research Building, North Bristol NHS Trust, Southmead Hospital, Bristol, BS10 5NB UK; 3 Faculty of Health Sciences, University of Southampton, University Road, Southampton, SO17 1BJ UK; 4 NIHR Bristol Biomedical Research Centre, University of Bristol, Level 3, University Hospitals Bristol Education Centre, Upper Maudlin Street, Bristol, BS2 8AE UK; 5 NHS Bristol, North Somerset and South Gloucestershire Clinical Commissioning Group, Brockway Medical Centre, Backwell and Nailsea Medical Group, 8 Brockway, Nailsea, Bristol, BS48 1BZ UK; 6 Population Health Sciences, Bristol Medical School, University of Bristol, Canynge Hall, 39 Whatley Road, Bristol, BS8 2PS UK; 7 Public and Patient Involvement Representative, Bristol, UK; 8 Translational Health Sciences, Bristol Medical School, University of Bristol, Bristol Urological Institute, Level 3 Learning and Research Building, Bristol, BS10 5NB UK

**Keywords:** TRIUMPH, Lower urinary tract symptoms, Cluster randomised controlled trial, Primary care, International Prostate Symptom Score

## Abstract

**Background:**

Lower urinary tract symptoms (LUTS) can relate to urinary storage or voiding. In men, the prevalence and severity of LUTS increases with age, with a significant impact on quality of life. The majority of men presenting with LUTS are managed by their general practitioner (GP) in the first instance, with conservative therapies recommended as the initial treatment. However, the provision of conservative therapies in primary care is variable and can be time and resource limited. GPs require practical resources to enhance patient engagement with such interventions. TRIUMPH aims to determine whether a standardised and manualised care intervention delivered in primary care achieves superior symptomatic outcome for LUTS versus usual care.

**Methods/design:**

TRIUMPH is a two-arm, cluster randomised controlled trial (RCT) being conducted in 30 National Health Service (NHS) general practices in England. The TRIUMPH intervention comprises a standardised LUTS advice booklet developed for the trial with patient and healthcare professional (HCP) consultation. The booklet is delivered to patients by nurses/healthcare assistants following assessment of their urinary symptoms. Patients are directed to relevant sections of the booklet, providing the manualised element of the intervention. To encourage adherence, HCPs provide follow-up contacts over 12 weeks. Practices are randomised 1:1 to either deliver the TRIUMPH intervention or a usual care pathway. The patient-reported International Prostate Symptom Score (IPSS) at 12 months post consent is the primary outcome. Secondary outcomes include cost-effectiveness, patient-reported outcomes on LUTS, quality of life, and patient and HCP acceptability and experience of the intervention. Primary analyses will be conducted on an intention-to-treat basis.

**Discussion:**

It is unclear whether conservative therapies for male LUTS are effectively delivered in primary care using current approaches. This can lead to men being inappropriately referred to secondary care or experiencing persistent symptoms. Primary care, therefore, holds the key to effective treatment for these men. The TRIUMPH intervention, through its standardised and manualised approach, has been developed to support GP practices in delivering effective conservative care. This pragmatic, cluster RCT should provide robust evidence in a primary-care setting to inform future guidelines.

**Trial registration:**

ISCRTN registry, ID: ISRCTN11669964. Registered on 12 April 2018.

**Electronic supplementary material:**

The online version of this article (10.1186/s13063-019-3648-1) contains supplementary material, which is available to authorized users.

## Background

For many men, lower urinary tract symptoms (LUTS) significantly affect quality of life (QoL) , work and other activities; such problematic LUTS are described as ‘bothersome’ according to the impact on the patient [1]. Since the risk of LUTS increases with age, the number of patients affected is likely to increase by almost 50% by the year 2025, in line with the ageing population [2].

LUTS can relate to storage (increased urinary frequency, nocturia, urgency, incontinence), voiding (slow stream, hesitancy, straining) or post-voiding (post-voiding dribble, sensation of incomplete emptying) symptoms. Men usually present with a range of LUTS. Particularly high-impact LUTS for men are; urgency/urgency incontinence, post-micturition dribble, nocturia and increased urinary frequency [3–5]. Disease-specific, health-related QoL measures are significantly worse in men with higher symptom frequency and severity ratings than in men with low symptom frequency and severity ratings in population-based studies [6]. LUTS can be caused by prostate enlargement leading to obstruction or bladder dysfunction, but are also influenced by men’s lifestyle and habits, such as overall fluid and caffeine intake.

The *UK National Institute of Health and Care Excellence (NICE) Clinical Guideline 97* [7] recommends that men receive key assessments (exclusion of serious medical conditions, malignancy and urinary tract infection), and assessment of the impact of their LUTS symptoms (storage/voiding/post voiding). Conservative treatment measures (fluid advice, bladder training, urethral compression and release, and pelvic-floor muscle exercises) are then recommended as initial interventions [7]. On the basis of a systematic review of assessment and therapy of male LUTS, the *European Association of Urology (EAU) Guidelines on Male LUTS for secondary care* stated that categorising precise symptoms (storage/voiding/post voiding) is an expectation of urological practice, and also recommended conservative treatment measures [8, 9].

The assessment expectations described in these guidelines are relatively time-consuming for a 10-min general practitioner (GP) consultation, with little resource to support the conservative approach. Thus, men may undergo limited assessment mainly to exclude serious underlying conditions. Ineffective delivery of conservative measures in primary care can mean that men simply receive a prescription of medication to treat the prostate, are inappropriately referred to secondary care or endure persistent symptoms. In a Quality and Productivity Proven Case Study the costs saved by reducing inappropriate referrals to secondary care for male LUTS were £21,652 per 100,000 population [10].

The evidence to support conservative interventions is limited. The Cochrane review on lifestyle interventions for the treatment of urinary incontinence in adults [11] suggested that there is insufficient evidence to justify fluid advice for treatment of urgency incontinence. However, an NHS Evidence Update indicated that self-management may have a role in the treatment of LUTS [12], citing a post-hoc analysis [13] of a single-centre randomised controlled trial (RCT) [14] of 140 men with LUTS assigned to a self-management programme plus standard care or standard care alone. Men assigned to the self-management programme reported better voided volumes, reduced daytime frequency and nocturia. The study had a relatively small patient population and was conducted in a single tertiary-treatment centre. The study did not affect NICE Clinical Guideline 97 [7] and indicated that a multicentre RCT would be needed to see if these results could be replicated in everyday clinical practice.

The aim of the TRIUMPH trial is to establish the clinical and cost-effectiveness of conservative therapies to treat men with LUTS through a manualised and standardised resource for primary care. The study has the potential merit of exploring the means to introduce self-management of LUTS into clinical care, and the primary-care setting reflects an NHS priority to reduce hospital referrals.

## Methods/design

### Aims and objectives

The key aim of this research is to determine whether a standardised and manualised care intervention achieves superior symptomatic outcome compared with usual care for male LUTS, with a primary outcome of overall International Prostate Symptom Score (IPSS) measured 12 months after consent, in a primary-care setting.

Secondary objectives are to compare the two trial arms with regard to:

1. Disease-specific QoL
2. Symptomatic outcomes
3. Cost-effectiveness
4. Relative harms
5. Use of NHS resources
6. Overall QoL and general health
7. Acceptability of assessment and provision of care
8. Patients’ perception of their LUTS condition

### Trial design

**Fig. 1:**
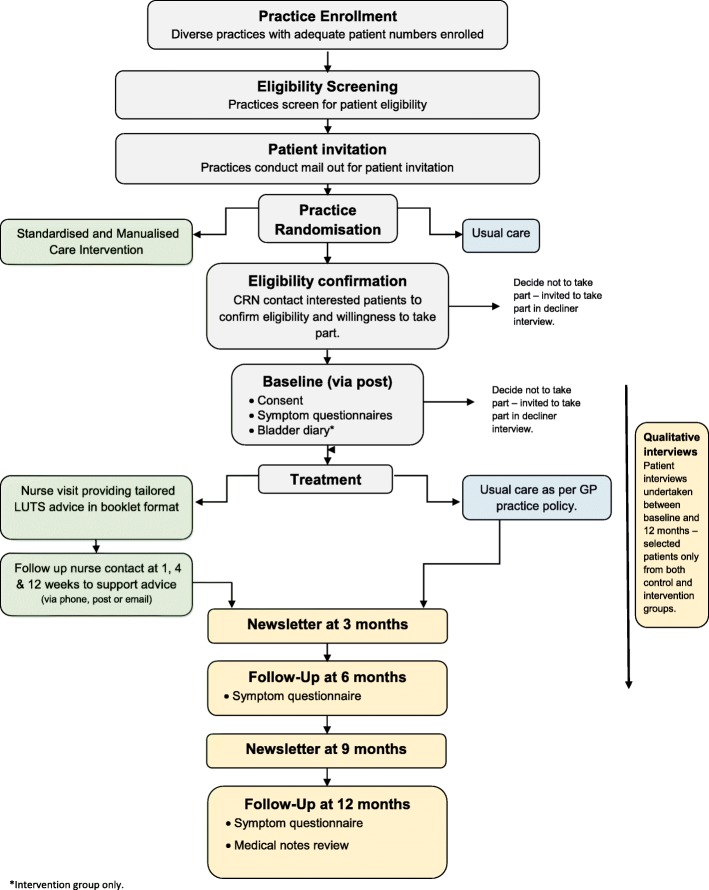
Study flow diagram

This is a pragmatic, two-arm, cluster RCT randomising GP practices 1:1 between a care pathway based on a standardised and manualised care intervention (intervention arm) and one based on usual care (comparator arm) for men with LUTS. The study flow diagram is provided in Fig. 1 and the Standard Protocol Items: Recommendations for Interventional Trials (SPIRIT) Checklist in Additional file 1. The trial design included an internal pilot recruitment phase of 4 months’ duration, primarily to verify that recruitment was possible before progression to the main phase of the trial.

The study is powered to detect a clinical improvement in overall IPSS score of 2. IPSS is a patient-reported questionnaire and will be completed at baseline, and at 6 and 12 months after consent, with the primary endpoint at 12 months post consent. Secondary outcomes are collected by questionnaire at 6 and 12 months and extracted from primary-care medical records at 12 months. All participants will be provided with study progress updates at 3 and 9 months via a newsletter to maintain engagement with the trial and encourage response rates of follow-up questionnaires. The newsletter does not provide any detail on the TRIUMPH intervention.

### Setting

Thirty GP practices were recruited within 12 CCGs across the West of England and Wessex Clinical Research Network (CRN) regions in the UK. Participants are identified and recruited from these practices.

### Recruitment of GP practices

The CRNs invited GP practices to express an interest in taking part in the study. In order to achieve a balanced range of practices, the following factors were considered:

- Number of potentially eligible patients, on conduct of a preliminary database search
- Patient list size
- Deprivation score (calculated using the GP practice postcode)
- Preference for intervention delivery (practice staff or trial research nurses (RNs))
- Treatment-room space available for intervention delivery

Selected sites underwent site initiation training. An internal pilot phase was conducted with eight initial sites over a period of 4 months before the main phase of the trial.

### Participants

This is a pragmatic trial, which includes all adult men who consider themselves to have bothersome LUTS, presenting to primary care within the past 5 years, with at least one symptom of LUTS. Only men with prevalent cases of LUTS are included in the trial. Detailed inclusion and exclusion criteria are provided in Table 1.

**Table 1:** Patient selection criteria

	Patient selection criteria
Inclusions	Adult men above the age of 18 years who have bothersome lower urinary tract symptoms (LUTS)
Exclusions	• Lack of capacity to consent • Unable to pass urine without a catheter (indwelling or intermittent catheterisation) • Relevant neurological disease or referral • Undergoing urological testing for LUTS • Currently being treated for prostate or bladder cancer • Previous prostate surgery • Poorly controlled diabetes mellitus • Recently referred or currently under urology review • Visible haematuria • Unable to complete assessments in English

### Patient screening and invitation

The process of patient invitation and screening is detailed below:

1. GP practices conduct a single database search to identify potentially eligible patients. The database search was developed specifically for the trial, based on the trial inclusion and exclusion criteria (Table 2) for both EMIS and SystmOne patient administration systems used in primary-care sites in both CRN regions
2. The patient list identified by the search is manually screened by GPs at the site against patient’s records using the eligibility criteria listed in Table 2, including criteria which could not be fully included in the search, such as lack of capacity to consent, and referral or review by secondary care
3. A de-identified screening log populated with eligibility codes for all patients identified from the search is sent to the central trial team
4. Invitation letters, with Patient Information Leaflets (PIL) and Expression of Interest (EOI) forms are mailed out to eligible patients by practices using an approved third party (Docmail – a secure service which automates sending invitation letters to potential participants). A single mail out is conducted for each site
5. Interested patients complete their EOI forms online or return paper copies by post
6. CRN nurses or clinical practitioners trained by the trial team conduct phone calls with interested patients. These calls are conducted whilst blinded to the allocation of the practice and, therefore, the patient, to avoid any bias. Calls are conducted to confirm eligibility, particularly the subjective criteria of whether the patient’s LUTS is bothersome to them, ensure patient understanding of the study, answer any questions and confirm willingness to participate

**Table 2:** Database search and manual screening criteria

	Database search
Inclusion criteria	
Adult men (age ≥ 18 years)	Male > 18 years old
Bothersome LUTS	LUTS; BPH; nocturia; urinary symptoms; prostatism; overactive bladder (within last 5 years)
Exclusion criteria	
Lack of capacity to consent	Dementia; learning disability; psychosis; schizophrenia (ever) – additional check in manual screen for any other indication of lack of capacity
Unable to pass urine without a catheter (indwelling or intermittent catheterisation)	Catheter code (in last 3 months)
Relevant neurological disease or referral	Dementia; Parkinson’s; MS; previous stroke (ever) Additional check in manual screen for any other neurological disease/referral that may affect LUTS
Undergoing urological testing for LUTS	Reviewed during manual screen only
Currently being treated for prostate or bladder cancer	Prostate or bladder cancer (ever)
Previous prostate surgery	TURP; prostatectomy; BNI (ever)
Poorly controlled diabetes mellitus	Latest HbA1c > 65
Recently referred or currently under urology review	Reviewed during manual screen only
Visible haematuria	Visible haematuria (in last 6 months)
Unable to complete assessments in English	Reviewed during manual screen only

*BNI* bladder neck incision, *BPH* benign prostatic hypertrophy, *HbA1c* glycosylated haemoglobin, *LUTS* lower urinary tract symptoms, *MS* multiple sclerosis

The number of patients manually screened by sites and included in the single mail out varied between the pilot and main phase of the trial:

- *Pilot phase*: during the pilot phase of the trial sites manually screened up to 325 potentially eligible patients identified from their database search, in order of NHS number. The maximum number of patients for an individual site to include in their invitation mail out was 150; however, the screening allowed for the potential of a second mail out. For sites with more than 150 eligible patients, the central study team randomly identified the 150 patients for invitation
- *Main phase*: during the main phase of the trial, sites with more than 220 eligible patients screen up to this number, in order of NHS number. The maximum number of patients invited into the trial from a single site is 220

### Cluster randomisation

GP practices are the units of allocation for the two study arms. Practices are randomised on a 1:1 basis to deliver either the TRIUMPH intervention or continue with usual care (control group) by a statistician blind to the identity of practices. Randomisation is conducted after the practices have completed their screening and invitation to eligible patients. Randomisation is minimised by centre (Bristol and Wessex), practice size (number of patients registered at the practice) and area-level deprivation (Index of Multiple Deprivation score, IMD) of the practice.

### Patient consent

**Fig. 2:**
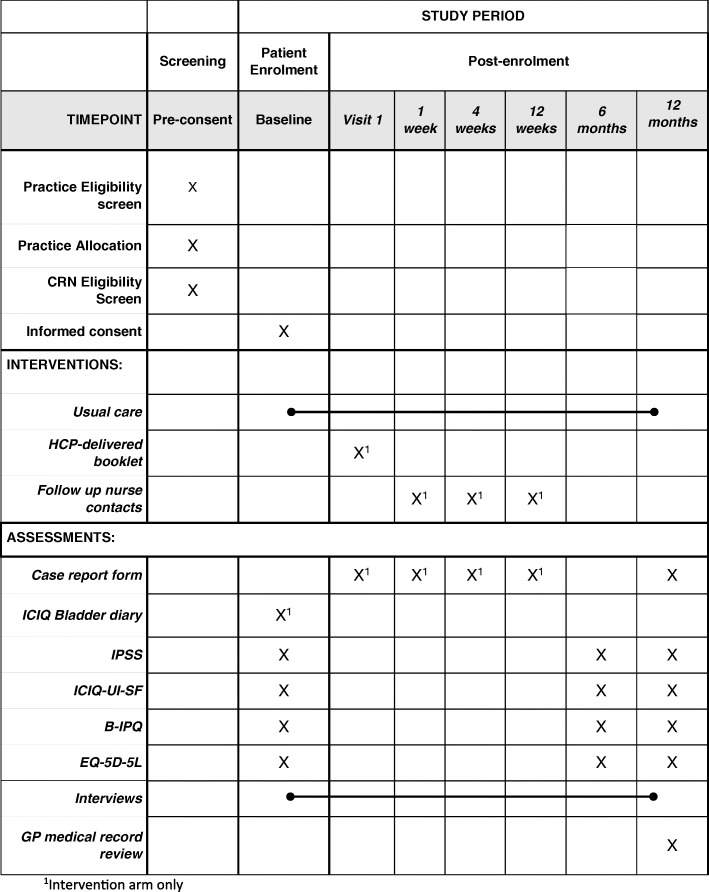
Schedule of enrolment, interventions and assessments

Patients deemed willing and eligible to participate in the study by the CRN are posted a consent form and a questionnaire containing baseline measures specific to trial arm for completion (Fig. 2). However, patients are blinded to their allocation until their completed questionnaire and consent form are received.

### Withdrawal

Participants remain in the trial unless they choose to withdraw, or if they are unable to continue. Patients may withdraw fully from the study or from specific elements without giving a reason. Any data collected up until the point of withdrawal are retained. Participants are informed of this in the PIL prior to consent.

### Blinding

Two statisticians are supporting this trial. The senior statistician co-applicant is blinded throughout the trial. The trial statistician will perform all disaggregated analyses according to a pre-specified statistical analysis plan and attends closed Data Monitoring Committee (DMC) meetings as required. The CRN support team is blinded whilst conducting patient eligibility calls to minimise selection and recruitment bias. Patients are blinded to their allocation until their completed baseline questionnaire and consent form are received. The trial health economist will remain blinded until completion of the cleaning of the resource-use data, and will perform all disaggregated analyses according to a pre-specified health economics analysis plan. The remaining members of the study team remain blinded to aggregate data only.

### Interventions

#### TRIUMPH intervention arm

LUTS presentations are typically a composite of different symptom combinations. The TRIUMPH intervention targets component symptoms with specific educational information and active management. This is provided in a standardised way in the form of a booklet which the patients can read in their own time to encourage take-up of the information. However, the intervention also provides manualised care, with a healthcare professional (HCP) using basic assessments and discussion with the patient to direct them to the most applicable information in the booklet. The discussion considers their personal circumstances, symptom needs, bothersomeness of these symptoms and impact on QoL. The TRIUMPH intervention arm, therefore, offers standardised and manualised care according to the symptomatic presentation of the individual patients.

The TRIUMPH intervention booklet underwent sequential development in multiple iterations and consultations involving patients, HCPs and health psychologists.

Details of the TRIUMPH intervention are provided below:

1. The intervention is delivered by a trained HCP; either a GP practice clinical nurse, RN or healthcare assistant (HCA), or dedicated trial RN depending on site preference
2. The HCP reviews the patient’s baseline urinary symptoms utilising their completed IPSS, The International Consultation on Incontinence Questionnaire Urinary Incontinence-Short Form (ICIQ-UI-SF) and ICIQ bladder diary
3. The participant attends for a single intervention visit, during which the HCP discusses their individual symptoms and level of bother. The HCPs are provided with a decision tool to assist them in tailoring the treatment for each patient at their intervention visit based on their symptoms
4. The HCP provides the patient with the illustrated booklet of written information ‘*Helping you to take control of your waterworks’*. The sections included are:
  Advice on drinks and liquid intake
  Advice on controlling an urgent need to urinate
  Exercising the muscles between the legs (pelvic floor) to help stop bladder leakage
  Advice on emptying the bladder as completely as possible
  Advice on getting rid of the last drops of urine
  Reducing sleep disturbance caused by needing to urinate

The booklet is water-resistant and able to lie flat when open, which is useful when potentially used when in the bathroom. Pictorial representations were used for clarity and avoid the use of potentially embarrassing images.

1. The delivery of the booklet is personalised by the HCP who directs the patient to the relevant sections of the booklet and, therefore, steps to take personally. A maximum of three sections are recommended to each patient and tabbed with discreet stickers. Where more than three relevant sections are identified as relevant to a patient, the three most bothersome are chosen. The choice of a maximum of three sections was guided by Patient and Public Involvement (PPI) consultation on what they considered to be a manageable level of advice to follow
2. To encourage and gauge adherence to the intervention, regular patient contact is provided following the initial face-to-face appointment. Follow-up contacts are conducted by phone at 1 week, and then by phone, email or text at 4 and 12 weeks according to patient preference. Patients retain the intervention booklet at the end of this period

The TRIUMPH intervention, therefore, aims to address key limitations in the current provision of care to men with LUTS in primary care through the use of symptom scores and bladder diaries to effectively diagnose specific LUTS symptoms, the provision of effective written materials, and training of RNs/HCAs in the interpretation of symptom scores and use of the Theory of Planned Behaviour to support self-management [15].

Patients in the intervention arm continue to receive usual care from their GP for their LUTS where necessary and details of this will be collected at 12 months.

#### Intervention training

The chief investigator (CI) developed the training and delivered this to the TRIUMPH study RNs. Materials were developed to support the training as well as the intervention visits. The TRIUMPH nurses attended GP practices to deliver training to practice nurses or HCAs. Training included examples of bladder diaries and how primary-care staff should interpret them for the purpose of the trial. Nurses were trained on the responses to the questionnaires and which sections of the booklet they should direct the participant to. This was supported by a checklist developed specifically for the study. Monthly teleconferences were held between the nurses/HCAs delivering the intervention and the trial managers for ongoing support, resolution of queries and sharing of experience.

#### Usual-care arm

Usual care (the comparator arm for TRIUMPH) in this study requests sites to continue to follow their standard local practice for trial patients. Usual care was chosen as the comparator for the study to reflect the actual care provided by control-arm GP practices rather than the National Institute for Health and Care Excellence (NICE) guidance which may be variably implemented. The qualitative aspect of this trial will explore what usual care looks like for a sample of comparator and intervention practices.

### Post-trial care

Following the end of the trial, patients in the intervention arm will be able to retain the booklet provided, and patients in the control arm will be provided with the booklet alongside a summary of the results of the trial. Their LUTS care will be the responsibility of their GP throughout the trial and after they have completed the TRIUMPH study at 12 months.

### Outcome measures

The primary outcome measure is the patient-reported IPSS at 12 months after consent. The IPSS is validated, extensively tested in LUTS research, and widely employed in urology services. The ICIQ-UI-SF supplements the IPSS with the inclusion of incontinence.

Secondary outcome measures:

- LUTS-specific QoL at 6 and 12 months (IPSS QoL)
- Symptoms scores at 6 months (IPSS overall score) and 6 and 12 months (ICIQ-UI-SF)
- Cost-effectiveness analyses from an NHS perspective with a 12-month time horizon. The EuroQol Group’s five-dimension health status questionnaire (EQ-5D-5 L) will be used to calculate quality-adjusted life years (QALYs)
- Number of adverse events (e.g. infection, urinary retention)
- Number of GP consultations
- Number of referrals to secondary care
- Overall QoL measured by the EQ-5D-5 L
- A qualitative element of the research study will evaluate patient experiences of the intervention
- Patient perception of their LUTS using the Brief Illness Perception Questionnaire (B-IPQ)

### Assessment and follow-up

The components and timing of follow-up measures are shown in Fig. 2.

#### Clinical and patient-reported outcomes

All participants complete self-reported outcome measures in the form of questionnaires (IPSS, ICIQ-UI-SF, EQ-5D-5 L and B-IPQ) at baseline (postal), 6 and 12 months (postal, online or phone) post enrolment. Participants are sent one reminder to return their baseline materials, and up to three reminders to return their 6- and 12-month questionnaires.

Data extraction of GP records at 12 months will be used to collect the secondary outcomes and healthcare resource measures including GP consultations, adverse events, referrals to secondary care and medications. Study-designed Case Report Forms (CRFs) are completed for the intervention arm only, at the intervention visit and during the 12-week treatment phase to collect details of the booklet sections advised to the patient, and feedback on the booklet.

#### Economic data collection

Data from the EQ-5D-5 L will be used to calculate quality-adjusted life years (QALYs). Information from the GP record data extraction will provide details of healthcare resource use for the economic evaluation. The intervention-arm CRFs will collect resources related to delivery of the intervention (e.g. HCP time).

#### Qualitative data collection

A qualitative component is included to evaluate patients’ attitudes to, and experiences of, standardised and manualised care interventions for men with LUTS, including patients within the control group and their LUTS experiences. Help-seeking drivers and previous experiences of LUTS management will also be explored. HCP views on the interventions will be explored as well as facets of trial participation. A small group of patients who decline to participate in TRIUMPH will be interviewed to explore their reasons for not participating and identify potential areas of improvement in the trial design.

Interviews will be semi-structured and follow a topic guide which will encourage participants to discuss their perspectives with regard to the aims above. They will be conducted during the pilot phase (control group excluded from this stage to explore acceptability of the intervention) and main phase with study participants. Interviews will focus on LUTS experiences and expectations from treatment at baseline. At 6–12 months following the treatment period, follow-up interviews will focus on outcomes and the experience of the treatments or interventions received in both groups. Clinicians will be interviewed to explore their perspectives on LUTS management, their perspectives on the intervention and standard care and their experience of being involved in the trial.

Interviews will be audio-recorded, transcribed verbatim and uploaded into a qualitative software package (NVivo10) to aid data management. Thematic content analysis will be undertaken on an ongoing basis in an iterative manner. Interviews will continue until data saturation is achieved in addition to ensuring representation of the different features of the trial and participants, such as age, presenting symptoms, geographical location and usage of different sections of the standardised booklet.

### Trial oversight

The study is supervised by a Trial Management Group (TMG) consisting of grant holders and other relevant trial delivery staff. A Trial Steering Committee (TSC) oversees the progress of the trial, comprising of an independent Chair and three other independent members, including a PPI representative, and the CI. A DMC monitors accumulating trial data for quality, completeness and patient safety, and comprises an independent Chair and two other independent members, and the CI. All serious adverse events (SAEs) are recorded and notified as appropriate to the relevant authorities.

### PPI

Extensive PPI was undertaken to develop the TRIUMPH intervention booklet, and also provided input on patient-facing materials and questionnaires. Patient representatives on both the TMG and TSC provide ongoing guidance for the trial, and with one patient as a co-applicant on the grant application, PPI input into the study has been ongoing since its conception. A wider Patient Advisory Group (PAG) also provide additional support.

### Data management and confidentiality

Study data are collected and managed using REDCap [16] hosted at the University of Bristol. The database incorporates data entry and validation rules to reduce data-entry errors, and management functions to facilitate auditing and data-quality assurance. The database system will protect patient information in line with the data protection legislation. Trial staff will ensure that participants’ anonymity is maintained through protective and secure handling and storage of patient information. The CI will have access to, and act as custodian of, the full dataset.

### Dissemination

The results of the study will be published in the academic press and all participants will be offered a lay summary of the main findings of the study. The trial will also be presented at national and international conferences. The results will be disseminated to the primary-care and urology communities through the relevant bodies.

### Sample size

A scoping search was conducted with local practices within the host CCG to gain a sense of the likely number of patients available on their practice lists based on the trial inclusion and exclusion criteria. This search suggested that an average-sized practice (7500) might identify 100 patients. Assuming that 50% of these patients will be eligible and 70% consent, each practice would consent 35 eligible patients. Our estimates of eligibility rates, consent and loss-to-follow-up are conservative and based on our experience running pragmatic trials.

This study is powered to detect a mean change of 2 points in IPSS scores at 12 months. The recognised minimum important difference in IPSS scores is 3 in men with LUTS generally [17], but a smaller difference was chosen in consultation with patient input when designing the TRIUMPH study because the study expects to include some men who have only one symptom requiring treatment (e.g. nocturia). We originally estimated that 840 patients are needed from at least 24 practices to detect a difference in IPSS scores of 2 (common standard deviation (SD) of 5; in line with the assumptions made in the UPSTREAM study [3]) with 90% power and significance level 5%. This estimate incorporated a design effect to account for clustering of effects in practices which assumed that practices will be able to recruit 35 patients each and that the intra-class correlation between practices would be 0.05 – an estimate in line with results from other primary-care studies [18]. We allowed for up to 30% of men being lost to follow-up.

During the pilot phase of the trial it became clear that the number of patients recruited by practices varied and that many were not recruiting the estimated 35 patients. Exploration of the recruitment data at practices that completed their mail outs and estimates for those to be opened suggested that 30 practices would be required to detect our stated effect size with 90% power assuming a mean practice size of 26 and coefficient of variation in practice size of 0.26.

### Statistical analysis

All analyses and reporting will be in line with Consolidated Standards of Reporting Trials (CONSORT) guidelines and its extension for cluster randomised trials. Primary analyses will be conducted on an intention-to-treat (ITT) basis. A full statistical analysis plan will be developed and agreed by the TSC prior to undertaking analyses of the main trial.

Descriptive statistics will be used to summarise characteristics of practices and patients and compare baseline characteristics between groups (age, deprivation based on practice location, height, weight, ethnicity and marital status). Means and SDs will be used for continuous and count outcomes or medians and interquartile range if required for skewed data. Categorical variables will be summarised using frequencies and proportions. Patient-reported outcome scores based on standardised questionnaires, including the primary outcome of LUTS score, will be calculated based on the developers’ scoring manuals and missing and erroneous items will be handled according to these manuals.

The primary outcome is the IPSS score collected at 12 months post baseline. It will be described in each treatment group using means and SDs. Comparisons between treatment arms will be made using a multilevel linear model to allow for clustering within practices adjusting for baseline IPSS scores and practice-level variables used in the randomisation. We will explore whether there is clustering in our primary outcome by the nurse/HCA delivering the intervention. If present, we will account for this in sensitivity analyses using a linear mixed model. The underlying assumptions of this model will be checked, and analyses adjusted accordingly.

Secondary outcomes explore LUTS, measures of QoL, self-management, adverse events, use of LUTS medication and referrals to primary and secondary care. Continuous outcomes will be studied in the same manner as the primary outcome using multilevel linear models to allow for clustering within practices adjusting for baseline measures of the outcome where available. Binary outcomes will be studied using multilevel logistic regression models allowing for clustering within practices. Count variables will be studied using multilevel Poisson regression models – or negative binomial model depending on the distribution of counts – allowing for clustering within practices. All models will adjust for variables used in the randomisation, the underlying assumptions of the models will be checked, and analyses adjusted accordingly.

### Economic data analysis

The trial will include a formal economic evaluation comparing the costs and cost-effectiveness of the intervention from an NHS perspective, from baseline to 12 months’ follow-up. The cost of the intervention and the use of primary and secondary NHS services by the men in relation to their bothersome LUTS will be estimated through the collection of resource-use data from GP records and study-designed proformas, and will be valued using routine data and GP practice information.

The values from EQ-5D-5 L, administered at baseline, 6 and 12 months, will be transformed into utility scores and individual QALYs will be calculated using the area-under-the-curve approach.

Resource use (e.g. number of GP consultations) will be calculated for each arm. Differences in costs and QALYs between the arms will be evaluated using appropriate regression techniques. For the primary economic analysis, cost-effectiveness will be assessed using the Net Benefit framework over a range of values for the QALY and will include the UK cost-effectiveness thresholds of £20,000–30,000.

A secondary economic analysis will examine the difference in costs and IPSS score. If neither arm is dominant (i.e. both cheaper and more effective), then an incremental cost-effectiveness ratio (ICER) will be calculated in relation to the IPSS score. If appropriate, Seemingly Unrelated Regressions (SUR) will be used when constructing the ICER, to account for the potential correlation between costs and the IPSS score.

Uncertainty for these analyses will be addressed using cost-effectiveness acceptability curves and sensitivity analyses.

### Qualitative data analysis

Theoretical purposive (non-probability) sampling will be used to ensure that the diverse characteristics of the population are sampled (e.g. participants of differing ages, clinical history, duration of symptoms and at follow-up in the intervention arm, components of the package received and drop-out/adherence). Geographical distribution will also be factored to ensure representation of varied practice populations [19]. Sampling and analyses will continue in iterative cycles until no new themes are emerging and established themes cease evolving (data saturation) [20]. It is anticipated that approximately 15 participants will be required for the feasibility stage, followed by a maximum of 30 patient interviews for both baseline and follow-up evaluation in the intervention arm and 15 at both time points in the usual-care arm during the main trial. Where possible we will conduct follow-up interviews with the same participants as the baseline interviews to capture reflective perspectives. However, additional participants may be required to ensure representativeness of the spectrum of interventions delivered and those considered compliant/adherent to the interventions.

A convenience sample of a maximum of 20 HCPs will also be interviewed to capture the variability of the practice populations and both usual care and intervention practices involved.

Analyses will be conducted by the qualitative researcher on an ongoing basis in an iterative manner, according to principles of thematic content analysis [21]. Recordings will be listened to and transcripts read and re-read for familiarisation. Segments of text will be ‘coded’ by assigning descriptive labels. Codes will be grouped on the basis of shared properties to create themes and coded transcripts will then be examined and compared to inductively refine and delineate themes (constant comparison) [22, 23].

A subset of interviews will be independently analysed by a second study researcher and coding discrepancies discussed to maximise rigour and reliability. Plausibility of data interpretation will be further discussed within the study team throughout the analyses. Descriptive summary accounts of the audio-recordings and interviews will be prepared.

## Discussion

This article outlines a multicentre, pragmatic, cluster RCT to compare standardised and manualised care versus usual care in primary care for men with bothersome LUTS. The aim is to determine whether the TRIUMPH intervention achieves superior symptomatic outcome compared with usual care for LUTS in primary-care setting, based on a patient-reported severity score (IPSS).

The internal pilot phase of the trial was successfully completed in November 2018. One hundred and forty-two participants were recruited in the 4-month pilot phase period, exceeding the target of 120 participants. Sixteen practices had formally agreed to take part in the study during the pilot phase which, although slightly short of the target of 18, was sufficient for progression to the main phase of the trial. The key changes that were made to the conduct of the trial between the pilot phase and the main phase of the trial were an increase in the maximum number of patients invited by a single site from 150 to 220, and confirmation that 30 sites would be required to maintain the trial power calculation in light of variation in the number of patients recruited per site. The decision was also taken to conduct only a single invitation mail out from each site to avoid introducing any bias post randomisation. Although screening of all patients for both mail outs would have been conducted before randomisation, re-screening would have been required to check patient status before a later second mail out, when sites would have been aware of their allocation.

This cluster randomised study should provide evidence in an NHS setting for the male LUTS population by using various patient-reported, clinical and cost-effectiveness outcomes to inform future NICE guidelines and potentially the opportunity to develop tools specifically for primary-care HCPs when treating this population. In the event that an HCP-led assessment and advice selection can reduce symptom severity, GP consultations in the future will be able to focus more on exclusion of serious conditions and potentially place less reliance on early drug prescription.

## Trial status

The TRIUMPH study commenced site recruitment in May 2018 and recruited the first patient on 10 July 2018. The internal pilot phase of recruitment was conducted over 4 months and completed on 10 November 2018. Recruitment will be completed at the end of July 2019. Protocol version 6.0, 4 April 2019.

## Additional file

Additional file 1: TRIUMPH Standard Protocol Items: Recommendations for Interventional Trials (SPIRIT) Checklist. (DOC 124 kb)

## Data Availability

Not applicable.

## Abbreviations

B-IPQ: Brief Illness Perception Questionnaire
BRTC: Bristol Randomised Trials Collaboration
CCG: Clinical Commissioning Group
CI: Chief investigator
CONSORT: Consolidated Standards of Reporting Trials
CRF: Case Report Forms
CRN: Clinical Research Network
DMC: Data Monitoring Committee
EAU: European Association of Urology
EOI: Expression of Interest
EQ-5D-5 L: EuroQol Group’s five-dimension health status questionnaire
GP: General practitioner
HCA: Healthcare assistant
HCP: Healthcare professional
HTA: Health Technology Assessment
ICER: Incremental cost-effectiveness ratio
ICIQ-UI-SF: International Consultation on Incontinence Questionnaire Urinary Incontinence-Short Form
IMD: Index of Multiple Deprivation score
IPSS: International Prostate Symptom Score
ISRCTN: International Standard Randomised Controlled Trial Number
ITT: Intention-to-treat
LUTS: Lower urinary tract symptoms
NHS: National Health Service
NICE: National Institute for Health and Care Excellence
NIHR: National Institute for Health Research
PIL: Patient Information Leaflet
PPI: Patient and Public Involvement
QALY: Quality-adjusted life year
QoL: Quality of life
RCT: Randomised controlled trial
SAE: Serious adverse event
SD: Standard deviation
SUR: Seemingly Unrelated Regressions
TMG: Trial Management Group
TSC: Trial Steering Committee
TURP: Transurethral resection of prostate
UK: United Kingdom
